# Disruption of the white matter structural network and its correlation with baseline progression rate in patients with sporadic amyotrophic lateral sclerosis

**DOI:** 10.1186/s40035-021-00255-0

**Published:** 2021-09-13

**Authors:** Wenbin Li, Qianqian Wei, Yanbing Hou, Du Lei, Yuan Ai, Kun Qin, Jing Yang, Graham J. Kemp, Huifang Shang, Qiyong Gong

**Affiliations:** 1 Huaxi MR Research Center (HMRRC), Department of Radiology, Functional and Molecular Imaging Key Laboratory of Sichuan Province, West China Hospital of Sichuan University, Chengdu, 610000 China; 2 Laboratory of Neurodegenerative Disorders, Departments of Neurology, West China Hospital of Sichuan University, Chengdu, 610000 China; 3 Department of Psychiatry and Behavioral Neuroscience, Division of Bipolar Disorders Research, University of Cincinnati College of Medicine, Cincinnati, OH 45267 USA; 4 Department of Musculoskeletal and Ageing Science and MRC - Versus Arthritis Centre for Integrated Research Into Musculoskeletal Ageing, Faculty of Health and Life Sciences, University of Liverpool, Liverpool, UK; 5 Research Unit of Psychoradiology, Chinese Academy of Medical Sciences, Chengdu, 610000 China

**Keywords:** Amyotrophic lateral sclerosis, White matter, DTI, Network, Connectomics, Machine learning, Psychoradiology

## Abstract

**Objective:**

There is increasing evidence that amyotrophic lateral sclerosis (ALS) is a progressive neurodegenerative disease impacting large-scale brain networks. However, it is still unclear which structural networks are associated with the disease and whether the network connectomics are associated with disease progression. This study was aimed to characterize the network abnormalities in ALS and to identify the network-based biomarkers that predict the ALS baseline progression rate.

**Methods:**

Magnetic resonance imaging was performed on 73 patients with sporadic ALS and 100 healthy participants to acquire diffusion-weighted magnetic resonance images and construct white matter (WM) networks using tractography methods. The global and regional network properties were compared between ALS and healthy subjects. The single-subject WM network matrices of patients were used to predict the ALS baseline progression rate using machine learning algorithms.

**Results:**

Compared with the healthy participants, the patients with ALS showed significantly decreased clustering coefficient *C*_p_ (*P* = 0.0034, *t* = 2.98), normalized clustering coefficient *γ* (*P* = 0.039, *t* = 2.08), and small‐worldness *σ* (*P* = 0.038, *t* = 2.10) at the global network level. The patients also showed decreased regional centralities in motor and non-motor systems including the frontal, temporal and subcortical regions. Using the single-subject structural connection matrix, our classification model could distinguish patients with fast versus slow progression rate with an average accuracy of 85%.

**Conclusion:**

Disruption of the WM structural networks in ALS is indicated by weaker small-worldness and disturbances in regions outside of the motor systems, extending the classical pathophysiological understanding of ALS as a motor disorder. The individual WM structural network matrices of ALS patients are potential neuroimaging biomarkers for the baseline disease progression in clinical practice.

**Supplementary Information:**

The online version contains supplementary material available at 10.1186/s40035-021-00255-0.

## Introduction

Amyotrophic lateral sclerosis (ALS) is an intractable progressive neurodegenerative disease characterized classically by neurodegeneration and loss of upper motor neurons of the corticospinal tract and lower motor neurons of the brainstem and spinal cord anterior horns [1]. While symptoms such as muscular weakness, spasticity and hyperreflexia may initially be manageable, the progressive loss of respiratory muscle innervation can lead to respiratory failure, typically within 2–4 years of symptom onset [2]. There is increasing evidence that ALS also affects multiple neural systems beyond the motor cortex and corticospinal tract [3] and there is an urgent need to identify reliable biomarkers for ALS progression in clinical practice and pharmacological trials [4].

Typically, decreased fractional anisotropy (FA) within focal brain regions including motor, frontal and prefrontal areas are found in white matter (WM) studies in ALS using diffusion tensor imaging (DTI) tractography [5] and tract‐based spatial statistics [6–8]. DTI metrics are sensitive markers for WM change [9], and have been recommended for ALS diagnosis [9] and assessment of disease progression [10, 11]. However, the direct correlations between focal magnetic resonance imaging (MRI) metrics and neuropsychological measures are questionable, because the motor, cognitive and behavioral functions are mediated by multisynaptic brain networks [12]. Therefore, the notion of selective anatomic vulnerability [13] is being supplemented and to some extent replaced by the syndrome-specific network vulnerability notion [14], which is supported by concepts such as network-wise degeneration [15], circuit-specific vulnerability [16] and disease progression along structural connectivity patterns [17]. DTI studies based on graph theory offer a valuable tool to analyze the topological organization of brain networks and inter-regional connections [18], which may be indicators of ALS progression [19]. In the ‘connectomics’ analysis, cortical and subcortical brain regions are parcellated into nodes, and the WM metrics of tracks between them taken as the edges of a mathematical graph. Such studies show that the brain networks have a “small-world” organization [18], intermediate between random networks, whose shorter overall path length is associated with a low level of local clustering, and regular networks or lattices, whose high level of clustering is accompanied by a long path length [18]. The small-world network architecture reconciles relatively independent functioning (i.e. segregation) with fast information transfer (i.e. integration) [20]. Application of this powerful approach to the brain structural and functional connectome in ALS has, however, yielded inconsistent results [19, 21–23].

Incorporating the interaction information across the whole brain, the single-subject network approach has value in characterizing objective brain features to distinguish patients from healthy individuals [24] as well as for predicting clinical outcomes after drug treatment [25]. Several clinical prognostic factors have been identified for ALS, including age, site of onset, functional and respiratory status, cognitive function, noninvasive ventilation, some genetic mutations [26], and clinical phenotypes [27]. In addition, there are some biological markers proposed as related to the ALS outcome, including dyslipidemia [28, 29], uric acid [30, 31], creatinine, albumin [4], and granulocyte count [32]. However, it is still unclear whether the WM network parameters have predictive value for the ALS baseline progression rate.

In the present study, we applied graph theoretical analysis to DTI data to compare the topological properties of brain WM networks between ALS patients and healthy controls (HC) at the global, regional and connection levels, and also evaluated the predictive value of the DTI-based connectome for the baseline ALS progression.

## Methods

### Participants

Seventy-three patients with ALS and 100 age- and sex-matched HCs were included in this study. All patients with ALS fulfilled the El Escorial revised criteria of the World Federation of Neurology for definite or probable ALS and none of them had affected family members. The severity of the disease was evaluated by the ALS functional rating scale-revised (ALSFRS-R). The exclusion criteria for patients were: (1) severe dysarthria and hand weakness; (2) meeting the criteria of ALS-frontotemporal dementia; and (3) a history of other neurologic conditions that could affect the assessment. The cognitive abilities of the ALS patients were estimated using the Addenbrooke’s Cognitive Examination–revised, Chinese Version. No patient showed significant cognitive impairment according to our prior studies [33, 34] at the time of their neuropsychological assessment. The ALSFRS-R assessed three latent domains corresponding to bulbar, motor and respiratory functions [35, 36], defined as follows: bulbar score = sum of ALSFRS-R questions 1–3 (maximum score 12); motor score = sum of ALSFRS-R questions 4–9 (maximum score 24); respiratory score = sum of ALSFRS-R questions 10–12 (maximum score 12) [35]. The baseline ALS progression rate was calculated as (48 – ALSFRS-R)/time since disease onset [37]. The disease onset time was obtained based on patients’ recall and cross-checked by at least one of the close family members or against medical records if available. The anxiety and depression level of patients was measured by the Hamilton Anxiety and Depression Rating Scale. Healthy participants were recruited from local community through poster advertisements. The exclusion criteria for all participants were: (1) presence of focal brain lesions on routine MRI; (2) claustrophobia or standard MRI incompatibility; (3) history of alcohol/substance abuse; (4) comorbidity with neurological or psychiatric disorders or serious physical disease (including traumatic brain injury, cerebrovascular disease, hypertension, diabetes mellitus, ischemic heart disease, chronic liver disease, or other chronic systemic disorders); and (5) poor image quality or severe head motion via visual inspection. This study was approved by the Human Research Ethics Committee of West China Hospital and written informed consent was obtained from all participants.

### Image acquisition

All participants were scanned using the same magnetic resonance scanner (3.0 T Siemens Trio, Erlangen, Germany) with a 12-channel head coil. Head motion was minimized by foam padding. DTI images were acquired using a spin-echo echo-planar sequence with the following parameters: 64 noncollinear diffusion directions with *b* = 1000 s/mm^2^ and a reference image without diffusion weighting (*b* value = 0), 3 mm slice thickness with no interslice gap, repetition/echo time (TR/TE) 6800/91 ms, field of view 1920 × 1920 mm^2^, flip angle 90°, voxel 0.94 × 0.94 × 3.0 mm^3^ and 2 excitations. High-resolution 3D T1-weighted images were acquired using a magnetization-prepared rapid gradient-echo sequence with the following parameters: resolution 1.0 mm × 1.0 mm × 1.0 mm, TR/TE 1900/2.26 ms, inversion time 900 ms; flip angle 9°, FOV 256 × 256 mm^2^, matrix size 256 × 256, slice thickness 1 mm, no interslice gap, voxel 1 × 1 × 1 mm^3^ and 176 slices.

### Image data processing and network construction

Data preprocessing and WM network construction were conducted mainly using the PANDA software (http://www.nitrc.org/projects/panda/; a pipeline tool for diffusion MRI analysis) [38]. The patient and control samples did not differ in scanning head motion for rotation, transition, and frame-wise displacement (all *P* > 0.05, Additional file 1: Table S1).

Whole-brain anatomical networks were constructed according to the approach used previously [39]. First, to define the nodes of the network, we used the automated anatomical labeling (AAL) atlas to divide the whole brain into 90 cortical and subcortical regions [40], as discussed and used previously [39, 41]. The connections between each pair of brain anatomical regions were determined by the FA value, which resulted in a 90 × 90 matrix for each participant. More details regarding the image processing and network construction work can be found in Additional file 2.

### Brain WM network topological measure analyses

We applied a network sparsity parameter, *S*, to give each network the same number of edges. According to previous studies [42, 43], we selected a range of *S* thresholds for the WM connectivity network such that: (1) the averaged degree over all nodes of each thresholded network was > 2 × log 90; and (2) the small‐worldness σ of all thresholded networks was > 1.1 in all participants. Based on these criteria, we defined *S* ranging from 0.1 to 0.34. For each network, the area under the curve (AUC), calculated over the range of *S* values with an interval step of 0.01, provides a summarized scalar for the topological characterization of brain networks unbiased by any single threshold.

Graph theoretical analysis was carried out on each participant’s WM network using the GRETNA software (http://www.nitrc.org/projects/gretna/) [44]. Both global (clustering coefficient *C*_p_, characteristic path length *L*_p_, normalized clustering coefficient *γ*, normalized characteristic path length *λ*, small‐worldness *σ*, local efficiency *E*_loc_ and global efficiency *E*_glob_) and regional metrics (nodal degree, betweenness and efficiency) were used to characterize network topology. For global measures, high values of *C*_p_, *γ*, and *E*_loc_ reflect network segregation, i.e., the ability for specialized neuronal processing carried out among densely interconnected regions; while low values of *L*_p_, *λ*, and high *E*_glob_ reflect network integration, i.e., the ability for global information communication or distributed network integration; *σ* characterizes an optimized balance between network segregation and integration [45, 46]. For nodal measures, the three kinds of nodal centrality measurements can reflect the topological importance of nodes in the network in different ways. More detailed explanation of topological measures can be found in the Additional file 2.

To detect the altered connectivity networks in patients with ALS, we used an NBS approach (http://www.nitrc.org/projects/nbs/) [47] to define a set of supra-threshold links in which any of the connected components and their sizes could be determined (threshold, *t* = 2.62, *P* < 0.05 equal to Cohen’s *d* = 0.2). The significance of each supra-threshold link among the connected components was estimated using a nonparametric permutation method (10,000 permutations).

### Statistical analyses

The demographic characteristics of the ALS and HC groups were compared using the R software (version 4.0.0). A nonparametric permutation test (repeated 10,000 times) was used to analyze between‐group differences in the AUC of global and nodal network metrics. After balancing statistical power against the risk of type I error, for all the nodal metrics, only the nodal centralities that changed in the same direction in at least two out of three different measures were reported [48]. After identification of the between-group differences in global and regional network metrics, partial Pearson’s correlation analyses were performed to assess their relationship with symptom severity including ALSFRS-R and its subscores, depression level and anxiety level, controlling for age, sex and illness duration.

### Prediction of the baseline progression rate using single-subject WM network

We further studied whether the single-subject WM network of patients with ALS can be used to predict their baseline progression rate. To increase the robustness of the prediction, the progression rate was binarized to “fast progression” and “slow progression” using a cut-off value of 0.68 per month based on previous studies [49, 50]. To reduce the feature dimension, we converted the raw connection data into principal components (PCs) using principal component analysis (PCA). We fed the PCs which explained 80% variance of the connection data into the linear kernel support vector machine (SVM) algorithm (Additional file 1: Fig. S1). To get the unbiased classification accuracy estimation and tune the hyperparameter C for the SVM, nested cross-validation was used (details found in Additional file 2).

To assess the significance of the prediction and to make sure that the proposed results did not reflect overfitting, we re-ran the study on a randomly permuted dataset. To do this, we shuffled the progression rate tags (slow and fast progression), breaking the relationship between ALS progression and MRI data and re-ran the analysis. This process was repeated for 5000 iterations and thus quantified the ability of the model to predict noise.

Finally, the top 10 PCs with highest weights in the SVM model were then mapped back from PCA space to WM connectivity space to identify the most important brain connections for ALS progression prediction. The predictor importance score for connections was defined as the product of the absolute value of the weight of the PC in SVM model and principal component scores of the connections [51].

## Results

### Demographic and clinical data

Demographic and clinical characteristics of the participants are summarized in Table 1. There were no significant differences between the two groups in age, sex or years of education (*P* > 0.05).

**Table 1:** Demographic and clinical characteristics of study participants

	ALS	CON	Statistical significance
Sample size	73	100	
Age, mean (SD), years	49.8 (7.9)	49.8 (8.7)	*t* = 0.98, n.s
Sex (female/male)	33/40	57/43	*χ*^2^ = 1.9, n.s
Education level, mean (SD), years	9.0 (3.2)	8.9 (4.1)	*t* = -0.16, n.s
BMI, mean (SD)	22.4 (2.7)	22.9 (2.8)	*t* = -1.3, n.s
Bulbar onset (%)	23.5%	n.a	
Disease duration, mean (SD), months	10.58 (5.86)	n.a	
Progression rate, mean (SD), units/month^a^	0.71 (0.63)	n.a	
Exposure to toxic substances (yes/no)^b^	15/58	n.a	
Anxiety level, mean (SD)^c^	4.1 (5.2)	n.a	
Depression level, mean (SD)^d^	7.2 (7.2)	n.a	
ALSFRS-R, mean (SD)	42.0 (3.9)	n.a	
ALSFRS-R_bulbar, mean (SD)^e^	10.8 (1.8)	n.a	
ALSFRS-R_motor, mean (SD)^f^	19.1 (3.5)	n.a	
ALSFRS-R_resp mean (SD)^g^	12 (0)	n.a	

*ALS* amyotrophic lateral sclerosis, *CON* healthy controls, *ALSFRS-R* Revised Amyotrophic Lateral Sclerosis Functional Rating Scale, *n.a.* not available, *n.s* not significant

^a^Calculated as (48 – ALSFRS-R score)/time since disease onset

^b^The toxic substances included pesticides, heavy metals, and organic solvents

^c^Anxiety level was evaluated by the Hamilton Anxiety Rating Scale

^d^Depression level was evaluated by the Hamilton Depression Rating Scale

^e^Bulbar score = the sum of ALSFRS-R questions 1–3 (maximum score of 12)

^f^Motor score = the sum of ALSFRS-R questions 4–9 (maximum score of 24)

^g^Respiratory score = the sum of ALSFRS-R questions 10–12 (maximum score of 12)

### Global and nodal topological alterations of the WM networks

**Fig. 1:**
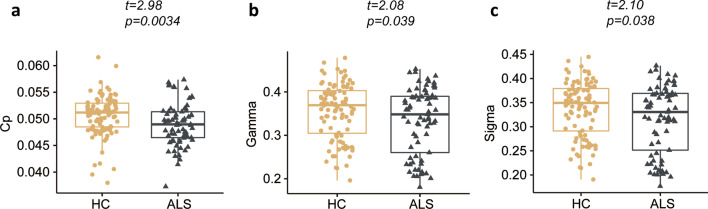
Abnormal global metrics in patients with ALS compared with HC. The clustering coefficients (**a**), normalized clustering coefficients (**b**) and small-worldness index (**c**) in ALS were significantly decreased compared with HC. Abbreviations: ALS, amyotrophic lateral sclerosis; HC, healthy controls; Cp, clustering coefficients; Gamma, normalized clustering coefficients; Sigma, small-worldness index

Both the ALS and the HC groups exhibited small-world properties of the WM structural network architecture, with *γ* > 1 and *λ* ≈ 1. The patients with ALS showed decreased *C*_p_ (*P* = 0.0034, *t* = 2.98), *γ* (*P* = 0.039, *t* = 2.08) and *σ* (*P* = 0.038, *t* = 2.10) compared with HC (Fig. 1). These differences were still significant after taking account of the outliers.

**Fig. 2:**
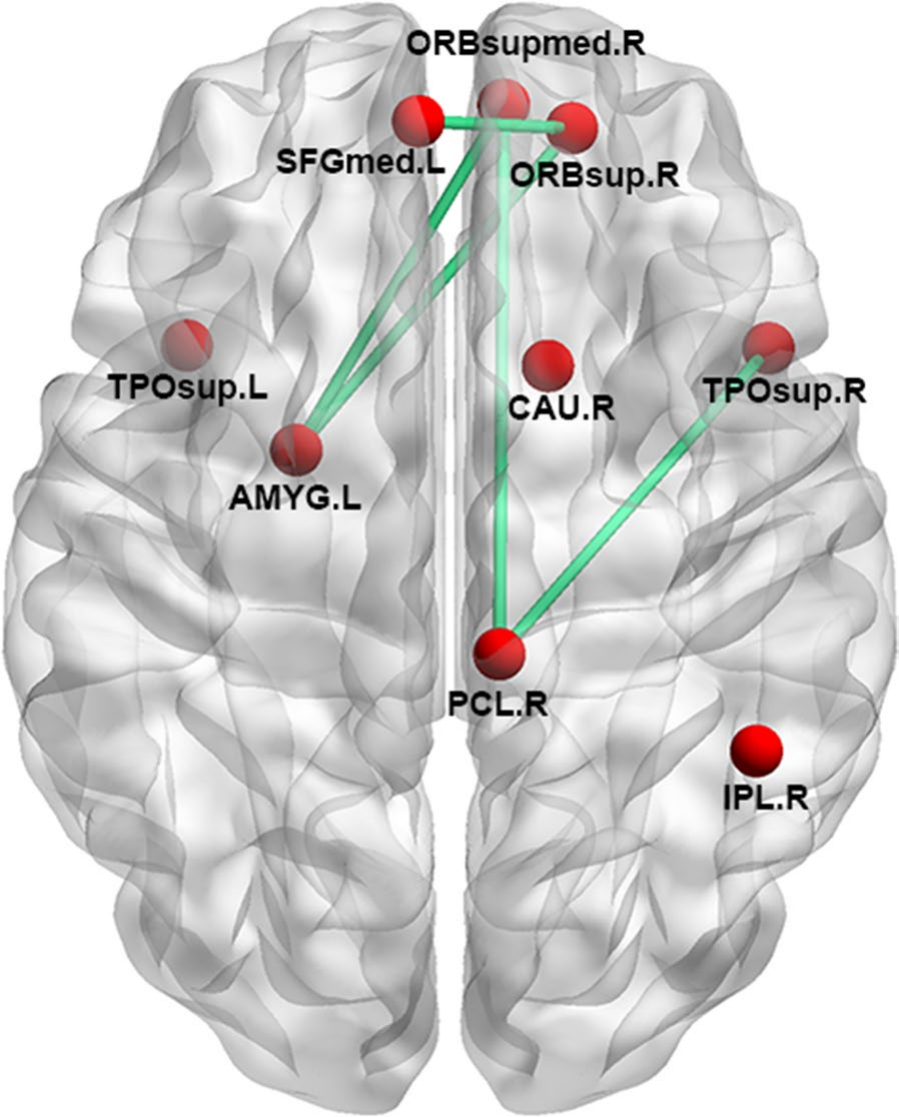
Nodal centrality and connection abnormalities in patients with ALS compared with the healthy controls. Nodal centralities in nine nodes (red) were decreased in ALS compared with the HC. Using NBS, 5 connections (green) were decreased in ALS compared with HC. SFGmed, superior frontal gyrus (medial part); TPOsup, temporal pole (superior part); AMYG, amygdala; IPL, inferior parietal lobe; CAU, caudate; ORBsupmed, orbital frontal cortex (superior medial part); ORBsup, orbital frontal cortex (superior part)

The nodal topological centralities were decreased in patients with ALS compared with the HCs in the right medial orbital frontal cortex, the left medial superior frontal cortex, the right gyrus rectus, the right paracentral lobule, the right inferior parietal cortex, the bilateral superior temporal pole, the left amygdala and the right caudate (*P* < 0.05, with significant change in the same direction in at least two of three centrality measures) (Fig. 2, Additional file 1: Table S2). Comparing the connections measured by FA values, we found a network less connected in ALS than in HC, with 6 nodes and 5 edges after NBS correction (Fig. 2).

Although the head motion parameters between patients and HCs were not significantly different (all *P* > 0.05, Additional file 1: Table S1), to further minimize the impacts of head motion on the primary results, we also re-ran the between-group comparisons taking all head motion parameters (including 3 translation, 3 rotation and 2 frame-wise displacement measures) as covariates. As expected, all between-group comparisons led to the same results as that obtained before.

### Relationships between topological properties and clinical variables

**Fig. 3:**
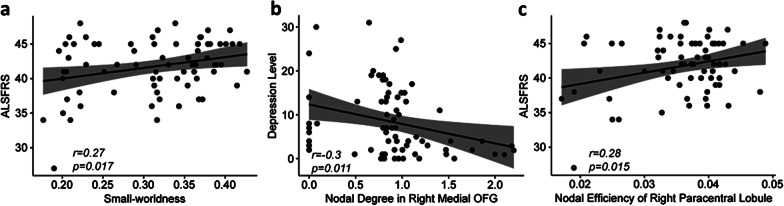
Correlations between topological metrics and clinical variables in patients with ALS. **a** Small-worldness indexes were positively correlated with ALSFRS. **b** Nodal degree centralities in right medial OFG were negatively correlated with the Hamilton depression scores. **c** Nodal efficiency centralities in right paracentral lobule were positively correlated with the ALSFRS. Abbreviations: ALS, amyotrophic lateral sclerosis; ALSFRS, amyotrophic lateral sclerosis functional rating scale; OFG, orbital frontal gyrus

Exploratory partial Pearson’s correlation analyses showed that the small-worldness indices were positively correlated with ALSFRS (*r* = 0.27, *P* = 0.017, Fig. 3a); the degree centrality of the right medial orbital frontal cortex was negatively correlated with depression symptoms (*r* = − 0.30, *P* = 0.011) (Fig. 3b) and the nodal efficiency of the right paracentral lobule was positively correlated with ALSFRS (*r* = 0.28, *P* = 0.015) (Fig. 3c). After excluding outliers, the correlation between the degree centrality of the right medial orbital frontal cortex and depression symptoms did not reach statistical significance (*r* = − 0.24, *P* = 0.051), but all other correlation results remained significant. We also found correlation relationships between network metrics and ALSFRS-R subscores (Table 2).

**Table 2:** Correlation between the network metrics and ALSFRS-R subscores

Network metrics	Class of subscores	*r* value	*P* value
C_p_	Motor	0.25	0.035
Nodal betweenness in R paracentral lobule	Bulbar	0.29	0.013
Nodal efficiency in R paracentral lobule	Bulbar	0.44	0.00013
Nodal efficiency in R caudate	Bulbar	0.24	0.046

*Cp* clustering coefficient, *R* right

### Machine learning analysis using single-subject WM networks to predict ALS progression

**Fig. 4:**
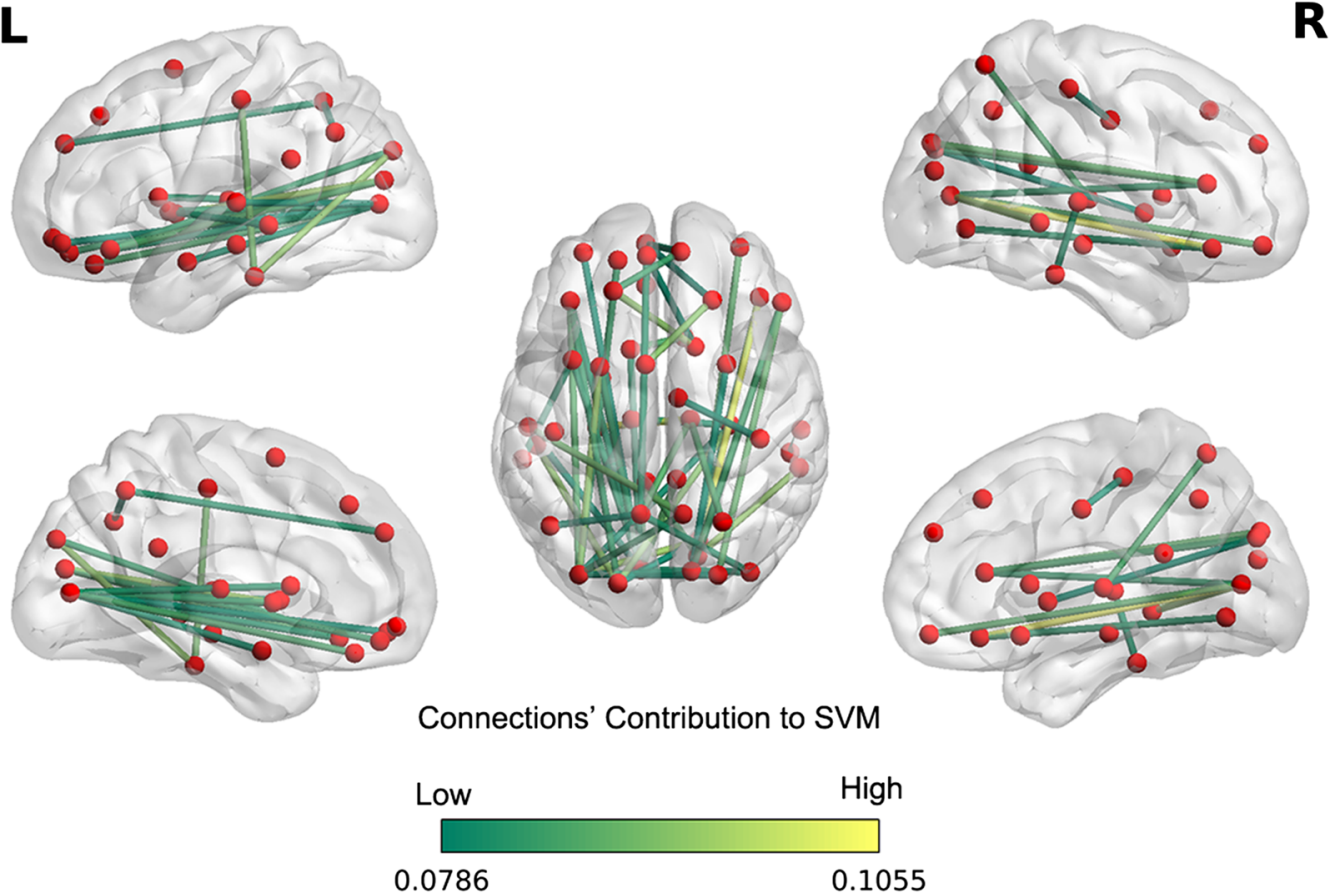
The connections which contributed most to the Support Vector Machine classification. Linear kernel SVM weights of the top 10 principal components were mapped back onto white matter connectivity data. Only the top 50 connections are shown: the color bar represents the predictive importance scores of connections in the WM network feature space. Abbreviations: L, Left; R, right

The demographic and head motion variables were comparable between the subgroups of fast and slow progression rate (Additional file 1: Table S3). Using WM matrices, the mean balanced classification accuracy for predicting ALS baseline progression rate was 85%, which is well above the chance expectation using the same model (*P* < 0.05, Additional file 1: Fig. S2). We calculated the predictive importance score for each connection in the WM network. The 50 most relevant WM connections contributing to the SVM classification are shown in Fig. 4 and Table 3.

**Table 3:** Top 50 relevant connections contributing to the SVM classification

White matter connection	Predictive weight
Hippocampus L to Thalamus R	0.105
Inferior orbital frontal gyrus R to Calcarine R	0.104
Lingual gyrus R to Middle occipital gyrus L	0.095
Middle occipital gyrus L to Putamen L	0.095
Calcarine R to Middle temporal gyrus R	0.094
Superior occipital gyrus L to Inferior temporal gyrus L	0.093
Superior frontal gyrus L to Caudate R	0.093
Hippocampus R to Superior occipital gyrus L	0.092
Superior frontal gyrus R to Supplemental motor area L	0.092
Postcentral gyrus L to Inferior temporal gyrus L	0.091
Precuneus R to Superior temporal gyrus L	0.090
Inferior frontal gyrus (triangular part) R to Superior occipital gyrus R	0.089
Posterior cingulum R to Calcarine L	0.088
Middle orbital frontal gyrus R to Calcarine R	0.087
Inferior orbital frontal gyrus L to Middle occipital gyrus L	0.087
Precuneus L to Thalamus R	0.087
Posterior cingulum L to Calcarine R	0.086
Olfactory R to Caudate L	0.086
Rectus L to Calcarine L	0.086
Superior parietal gyrus R to Thalamus R	0.086
Inferior frontal gyrus (triangular part) R to Cuneus R	0.085
Superior frontal gyrus L to Medial superior frontal gyrus R	0.084
Superior orbital frontal gyrus L to Middle occipital gyrus L	0.084
Inferior orbital frontal gyrus L to Superior occipital gyrus L	0.084
Middle occipital gyrus L to Precuneus R	0.084
Calcarine L to Putamen L	0.084
Inferior frontal gyrus (triangular part) R to Calcarine R	0.083
Insula L to Calcarine L	0.083
Inferior orbital frontal gyrus R to Lingual R	0.082
Calcarine L to Caudate L	0.082
Superior temporal gyrus R to Inferior temporal gyrus R	0.082
Middle occipital gyrus R to Precuneus L	0.082
Medial superior frontal gyrus L to Precuneus L	0.081
Inferior orbital frontal gyrus L to Calcarine L	0.081
Insula L to Middle temporal gyrus L	0.081
Calcarine L to Middle occipital gyrus R	0.081
Superior medial frontal gyrus R to Medial orbital frontal gyrus L	0.081
Hippocampus R to Thalamus L	0.081
Cuneus R to Occipital Mid L	0.080
Amygdala L to Calcarine L	0.080
Hippocampus R to Occipital Mid L	0.079
Middle cingulum R to Postcentral gyrus R	0.079
Angular L to Precuneus L	0.079
Medial superior frontal gyrus L to Caudate R	0.079
Superior occipital gyrus R to Middle occipital gyrus L	0.079
Middle orbital frontal gyrus L to Calcarine L	0.079
Lingual R to Superior occipital gyrus L	0.079
Calcarine L to Superior temporal gyrus L	0.079
Superior frontal gyrus R to Medial orbital frontal gyrus L	0.079
Cuneus R to Putamen R	0.079

*L* left, *R* right

## Discussion

In the present study, we found significant changes in the topological architecture of brain structural network at different levels. At the global level, the whole‐brain WM network showed decreased small-worldness in patients with ALS, reflected by lower *σ*, and decreased segregation reflected by lower *C*_p_ and *λ*. At the regional level, several nodes located mainly in the frontal, temporal and subcortical regions showed decreased topological centralities in patients with ALS. At the connection level, we found decreased WM connections between the nodes with decreased centralities. In addition, the machine leaning model showed that the single-subject structural connection network can be used as a biomarker to predict the ALS progression rate, which may inspire further clinical practice.

The WM structural networks in patients with ALS showed weaker small-worldization, evidenced by decreased clustering coefficients and small-worldness index. The small‐world organization reflects an optimal balance between network segregation (reflected by *C*_p_, *γ*, or *E*_loc_) and network integration (reflected by *L*_p_, *λ*, or *E*_glob_) of information processing [20], and the balance can be measured as *σ* [52]. Despite having an overall small‐world architecture qualitatively similar to HC, patients with ALS showed lower *C*_p_ and *γ*, resulting in a lower small-worldness index *σ*. These alterations of small-worldness were positively correlated with ALSFRS, suggesting a clinical relevance.

These results are consistent with a recent multicenter study that reported altered global structural brain network properties in patients with ALS [53]. In contrast, several early studies on WM connectomics have reported no global topological alterations in patients with ALS [21, 22, 54]. Although these studies utilized similar MRI sequences and tracking methods, their sensitivity to these topological changes may have been limited by relatively small sample sizes and/or relatively low numbers of non-collinear diffusion directions in the DTI sequence. Zhang et al. have reported that patients with ALS show a consistent rearrangement towards a regularized architecture evidenced by increased path length and clustering coefficient [23]. This difference may result from different network definitions, as they used the structural covariance networks, in which the connections were defined by the Pearson correlation coefficients between two regions of interest in gray matter. However, rearrangement toward a regularized network itself reflects a breakdown of the original optimal small-world network architecture. No doubtfully, different modalities can provide different perspectives on network abnormalities. In future connectome studies on ALS, different modalities can be combined to include functional MRI, diffusion MRI and gray matter MRI in larger sample sizes.

In addition to the global topological abnormalities, we found topological alterations in several brain regions. Consistent with the recent multicenter study [53], we found decreased nodal centralities in ALS patients in both motor and nonmotor networks including the secondary motor regions, the prefrontal regions, the temporal regions (superior temporal pole), the basal ganglia regions and the parietal region.

The cortical motor system is a distributed network of areas involved in different aspects of specific motor execution. Even simple movements are associated with activation of multiple cortical areas of the primary motor cortex and the secondary motor regions [55–57], including the supplementary cortex, the premotor cortex, the paracentral lobule and the superior parietal motor areas, which are highly inter-connected, converging on the primary motor cortex [58]. Our results suggest that deficits of the secondary motor regions may be an important trait in ALS. Consistent with this, earlier neuroimaging studies have reported decreased cortical thickness and gray matter volume of the secondary motor regions in patients with ALS [59–61].

The pathological hallmarks of ALS are tau-negative and ubiquitin-positive intraneuronal inclusions, and the 43-kDa TAR DNA-binding protein (pTDP-43) is a major component of the inclusions specific for frontotemporal lobar degeneration and ALS [62]. Initially, the TDP-43 burden is greatest in the agranular motor cortex and brainstem motor nuclei [63]. As the disease progresses, the pTDP-43 lesions increasingly involve the prefrontal (gyrus rectus and orbital gyri), striatum, amygdala and temporal lobe along axonal pathways [63, 64]. Consistently, neuroimaging studies in ALS have also confirmed the spread of atrophy and/or hypometabolism to the frontal and temporal cortices [65–67]. DTI studies have also reported WM deficits in the frontotemporal regions [68, 69]. Longitudinal and combined structural and functional MRI studies are needed to validate our hypothesis of disease progression along the functional and structural connections of the frontotemporal network.

We also found that the single-subject networks can predict the disease progression rate with an accuracy of 85%. Earlier studies also found that MRI abnormalities can be used to predict outcome in ALS. More severe abnormalities of the corticospinal tract and the spinal cord predict a poorer long-term clinical outcome in patients with ALS [70, 71]. FA has been proven to be a sensitive DTI metric for both diagnosis [72] and progression modeling [73]. An earlier WM network study has also found a relationship between the FA-based connectivity degree in the frontal area and disease progression rate of patients with ALS [19]. Similarly, using deep learning, van der Burgh and colleagues also predicted outcomes of ALS patients with high accuracy by combining the WM network, morphology and clinical information [74]. As an important alternative approach to studying ALS pathology progression, earlier studies [75–78] also found that alterations of network and other imaging features provide useful information associated with disease progression [79] in the spatial domain. All this evidence indicates that the brain network information has significant predictive potential to predict disease progression in patients with ALS as a supplement to other clinical measures.

The study has several limitations. First, although the method for echo plane imaging-distortion correction (i.e. non-linear registration) used in the current study is common in the field, state-of-the-art distortion correction methods like file-mapping [80], *topup-*based approach [81–83], or machine learning approaches [84, 85] are encouraged to be used in future studies. Second, currently there is no widely accepted optimal approach to defining nodes and edges. We used the widely used AAL 90 template regions as nodes and mean FA values of fibers as the weighting factor in the construction of graphs. Other measures such as Harvard–Oxford atlas can also be considered for calculating network metrics [38]. Third, this study was cross-sectional; how the WM network architecture associated with ALS evolves dynamically and how the WM network evolves in the progress of disease remain to be clarified in longitudinal studies. Fourth, in this study the progression rate was based on a retrospective interview. As ALS progression is dynamic, a prospective study design would be more suitable for the prediction analysis. Fifth, we did not collect genetic information of the ALS patients. Although genetic factors have less impact on sporadic ALS compared with familial ALS [86], further studies should also collect the genetic information to study the interactions between MRI and genetic information. Finally, to reduce the scanning time and thus limit obstacles to participation, we chose 3-mm slice thickness DWI. This has led to non-isotropic voxels which might have a negative effect on the FA estimation and tractography. High-resolution diffusion-weighted images with isotropic voxel size would be a better choice in future studies, if scanner timing permits.

## Conclusion

Our study demonstrated disruption of the WM structural networks in ALS, indicated by weaker small-worldness and regional disturbance in the regions outside of the motor systems, which might extend our typical understanding of ALS as a motor disorder; further, the WM structural network has potential to serve as the neuroimaging biomarkers for predicting the progression of ALS. This study also adds to the field of psychoradiology [87–89], an evolving subspecialty of radiology, which is primed to be of major clinical importance in guiding diagnostic and therapeutic decision-making in patients with neuropsychiatric disorders.

## Supplementary Information

**Additional file 1: Table S1**. Head motion metrics in the participants. **Table S2**. Nodal topological metrics showing differences between ALS patients and healthy controls. **Table S3**. Demographic and head motion variables of two subgroups with different progression rate. **Figure S1**. Scree plot of the proportion of variance explained by each principal component. **Figure S2**. Classification performance for real and permutated data

**Additional file 2**. Materials and Methods

## Data Availability

The data that support the findings of this study are available from the corresponding author upon reasonable request. The data and code sharing adopted by the authors comply with the requirements of the funding institute and comply with institutional ethics approval.

## Abbreviations

AAL: automated anatomical labelling
ALS: amyotrophic lateral sclerosis
ALSFRS: amyotrophic lateral sclerosis functional rating
AUC: area under the curve
DTI: diffusion tensor imaging
FA: fractional anisotropy
MRI: magnetic resonance imaging
PCA: principal component analysis
PC: principal component
pTDP-43: 43-KDa TAR DNA-binding protein
SVM: support vector machine
WM: white matter
