# Supplementary material for: Disruption of the white matter structural network and its correlation with baseline progression rate in patients with sporadic amyotrophic lateral sclerosis
Source: Transl Neurodegener. 2021 Sep 13;10:35. doi: 10.1186/s40035-021-00255-0 (PMC8436442; doi:10.1186/s40035-021-00255-0)
Supplement: Supplementary file 1 — Additional file 1: Table S1. Head motion metrics in the participants. Table S2. Nodal topological metrics showing differences between ALS patients and healthy controls. Table S3. Demographic and head motion variables of two subgroups with different progression rate. Figure S1. Scree plot of the proportion of variance explained by each principal component. Figure S2. Classification performance for real and permutated data [file 40035_2021_255_MOESM1_ESM.docx]

## Additional file 1

## Table S1. Head motion metrics in the participants

|  | Patients with ALS (*n* = 73) | HC (*n* = 100) | Statistics |
| --- | --- | --- | --- |
| Translation in x axis (mm) | 0.007 (0.37) | -0.14 (0.77) | *t* = 1.59, *p* = 0.11 |
| Translation in y axis (mm) | 0.67 (0.17) | 0.71 (0.25) | *t* = -1.21, *p* = 0.23 |
| Translation in z axis (mm) | 0.53 (0.52) | 0.48 (0.46) | *t* = 0.58, *p* = 0.57 |
| Rotation in x axis (radiance) | -0.0007 (0.01) | -0.0001 (0.0082) | *t* = -0.39, *p* = 0.70 |
| Rotation in y axis (radiance) | -0.00017(0.0058) | 0.0018 (0.0093) | *t* = -0.7, *p* = 0.09 |
| Rotation in z axis (radiance) | 0.000055 (0.008) | -0.003 (0.013) | *t* = 1.71, *p* = 0.09 |
| Absolute FD (mm) | 1.86 (0.53) | 1.99 (0.86) | *t* = -1.27, *p* = 0.20 |
| Relative FD (mm) | 0.47 (0.30) | 0.39 (0.26) | *t* = 1.83, *p* = 0.07 |

Abbreviation: FD, frame-wise displacement.

## Table S2. Nodal topological metrics showing differences between ALS patients and healthy controls

| Graph metrics | Nodal Degree | | Nodal Betweenness | | Nodal Efficiency | |
| --- | --- | --- | --- | --- | --- | --- |
|  | ALS | HC | ALS | HC | ALS | HC |
| Right medial frontal cortex (orbital part) | 0.80 (0.49) * | 0.95 (0.39) | 1.51 (3.00) | 1.15 (2.80) | 0.034 (0.017) ** | 0.040 (0.010) |
| Left medial superior frontal cortex | 1.01 (0.49) * | 1.20 (0.49) | 7.72 (7.83) ** | 11.17 (9.44) | 0.039 (0.020) * | 0.043 (0.011) |
| Right gyrus rectus | 0.63 (0.39) * | 0.75 (0.34) | 1.86 (2.61) * | 3.08 (3.92) | 0.032 (0.016) ** | 0.037 (0.010) |
| Right paracentral lobule | 0.63 (0.30) | 0.71 (0.31) | 0.77 (1.25) ** | 1.55 (2.29) | 0.036 (0.0064) ** | 0.039 (0.0050) |
| Right inferior parietal cortex | 0.60 (0.36) ** | 0.83 (0.55) | 0.88 (1.77) ** | 2.06 (3.92) | 0.036 (0.0062) | 0.038 (0.0060) |
| left superior temporal pole | 0.83 (0.47) ** | 1.01 (0.41) | 3.35 (4.97) | 4.32 (4.27) | 0.035 (0.016) * | 0.040 (0.010) |
| Right superior temporal pole | 0.72 (0.43) * | 0.85 (0.37) | 3.10 (4.22) | 3.82 (4.46) | 0.033 (0.016) * | 0.037 (0.011) |
| Left amygdala | 0.51 (0.32) | 0.59 (0.29) | 0.71 (1.52) ** | 1.29 (2.18) | 0.033 (0.014) * | 0.037 (0.0099) |
| Right caudate | 1.25 (0.47) ** | 1.49 (0.54) | 9.81 (8.17) *** | 17.06 (13.48) | 0.043 (0.010) * | 0.047 (0.0086) |
| Data shown as mean (s.d.). Abbreviations: ALS, Amyotrophic lateral sclerosis; HC, healthy controls; * *p* <0.05; ** *p* <0.01; *** *p* <0.001 | | | | | | |

## Table S3. Demographic and head motion variables of two subgroups with different progression rate

|  | Fast Progression (*n* = 26) | Slow Progression (*n* = 47) | Statistical Significance |
| --- | --- | --- | --- |
| Age, mean (SD), years | 48.42(7.92) | 50.57(7.84) | *t* = -1.11, n.s. |
| Sex (female/male) | 9/17 | 24/23 | *χ^2^* = 1.22, n.s. |
| Education level, mean (SD), years | 8.20(2.65) | 9.40(2.83) | *t* = -1.66, n.s. |
| BMI, mean (SD) | 22.34(2.31) | 22.67(2.28) | *t* = -0.59, n.s. |
| Translation in x axis (mm) | 0.056(0.28) | 0.01(0.39) | *t* = 0.57, n.s. |
| Translation in y axis (mm) | 0.68(0.17) | 0.67(0.16) | *t* = 0.21, n.s. |
| Translation in z axis (mm) | 0.62(0.44) | 0.49(0.58) | *t* = 1.06, n.s. |
| Rotation in x axis (radiance) | -0.00071(0.0068) | -0.0012(0.012) | *t* = 0.20, n.s. |
| Rotation in y axis (radiance) | -0.0014(0.0056) | 0.00042(0.0056) | *t* = -1.34, n.s. |
| Rotation in z axis (radiance) | 0.00080(0.0055) | -0.00014(0.0093) | *t* = 0.54, n.s. |
| Absolute FD (mm) | 1.84(0.37) | 1.91(0.60) | *t* = -0.62, n.s. |
| Relative FD (mm) | 0.51(0.36) | 0.46(0.28) | *t* = 0.57, n.s. |
| Abbreviations: BMI, Body Mass Index; FD, Frame-wise Displacement; n.s, not significant (*p* > 0.1). PR, Progression Rate. | | | |


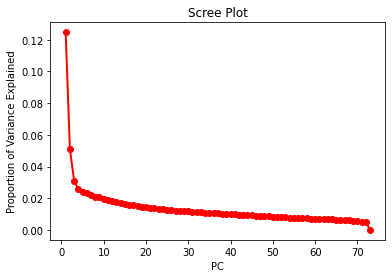


**Figure S1**. Scree plot of the proportion of variance explained by each principal component. A scree plot for explained variance for the 73 principal components (PCs) from the principal component analysis (PCA). The X-axis displays the PCs and the Y-axis on the left shows percentage of variance explained. 45 PCs containing 80% variance of the data were included in further analysis.


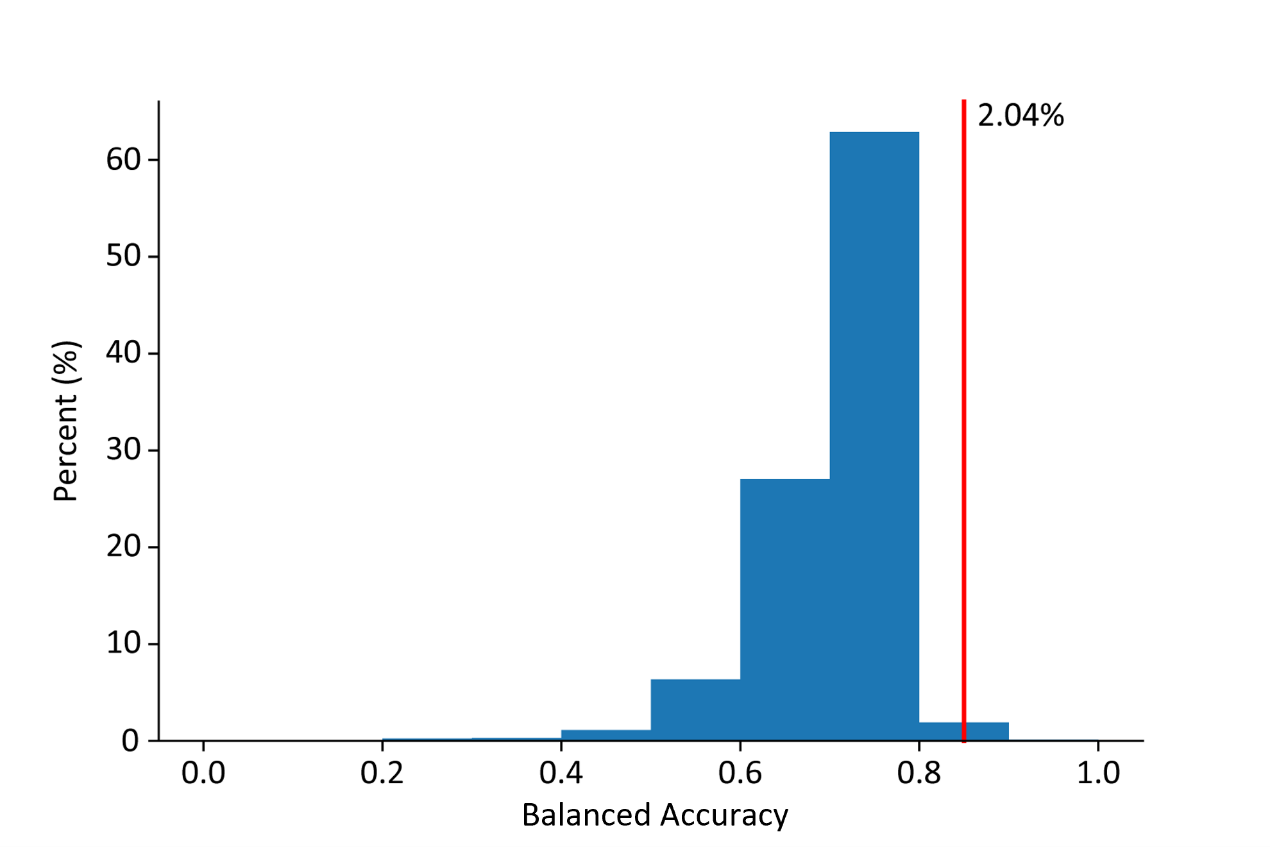


## Figure S2. Classification performance for real and permutated data. Cross-validated classification performance (balanced accuracy) in classifying fast vs. slow baseline ALS progression. The red line depicts the accuracy for the real data, and the histogram shows the model’s performance in predicting shuffled data (random permutation). The % value next to the red line shows the likelihood that the model performance can be achieved by chance.
