# Supplementary material for: Disruption of the white matter structural network and its correlation with baseline progression rate in patients with sporadic amyotrophic lateral sclerosis
Source: Transl Neurodegener. 2021 Sep 13;10:35. doi: 10.1186/s40035-021-00255-0 (PMC8436442; doi:10.1186/s40035-021-00255-0)
Supplement: Supplementary file 2 — Additional file 2. Materials and Methods [file 40035_2021_255_MOESM2_ESM.docx]

## Additional file 2

## MRI preprocessing and single-subject WM network construction

Diffusion weighted (DW) image preprocessing was performed as follows. First, DICOM files were converted into NIfTI images using the *dcm2nii* tool embedded in MRIcorn. The brain mask for each was estimated using the *bet* command of FSL. The raw images were cropped: brain borders along each dimension were determined by the brain masks (which reduces memory cost and speeds processing), and the *fslroi* command of FSL was used to remove the non-brain spaces. Correction for head-motion and eddy-current artifacts was performed by registering the DW images to the b_0_ image with an affine transformation, using the *eddy_correct* command. EPI-(echo-planar imaging) distortion unwarping was performed by nonrigid registration of each participant's DWI data to their T1-weighted image, then non-linear registration of each structural image to Montreal Neurological Institute (MNI) space. Based on the transformations in these two steps (EPI to T1-weighted image, then T1-weighted image to MNI), an inverse warping transformation was obtained from the standard space to the native diffusion MRI space, and used to inversely warp each AAL atlas in the standard space to the individual native diffusion space, preserving the discrete labeling values using nearest-neighbor interpolation. The transformed AAL atlas and the b_0_ image for each participant were visualized together in the individual space and checked by two of the authors (WL and DL) independently to ensure exclude obvious mismatches.

The preprocessed DWI images were used to build diffusion tensor models and obtaining fractional anisotropy (FA) maps with DTIFIT. Deterministic fiber tracking (chosen because it yields more accurate connectome reconstructions) [1] was implemented with Diffusion Toolkit (http://trackvis.org/dtk/), using the Fiber Assignment by Continuous Tracking (FACT) algorithm. Deterministic fiber tractography proceeded until either it turned an angle > 45° or the FA was < 0.2 [2, 3].

Network construction was then based on fiber tracking results. Nodes were defined using the parcellation of AAL in the native diffusion space. Next, to define the edges of the network, the averaged FA of linking fibers was calculated between each pair of nodes, from was obtained a weighted and undirected symmetrical anatomical 90 × 90 matrix for each participant.

## Brain topological measures

Global metrics, which measure the architecture of the whole brain network, included: small‐world parameters (clustering coefficient C_p_, characteristic path length L_p_, normalized clustering coefficient γ, normalized characteristic path length λ, and small‐worldness σ) and network efficiency parameters (local efficiency E_loc_ and global efficiency E_glob_). Nodal metrics, which measure the topology of single network nodes, included three nodal centrality metrics: nodal degree [4], nodal efficiency [5] and nodal betweenness [6]. The most important network complexity measure is node degree since many other graph theory measures are related to it. The degree of a node equals to the number of edges connected to it. It reflects its information communication ability in the network. Betweenness centrality is defined as the fraction of all shortest paths in the network that pass-through a given node, reflecting the nodes’ effects on information flow between other nodes. The efficiency of a node defined as the inverse of the shortest path length between one node and other nodes measuring how efficient information is transferred between this node and the others. The smaller the distance between nodes, the faster the information transfers. The average efficiency of all nodes in a graph is called E_g_. Similarly, the average of shortest path length of all nodes in a graph is called L_p_. Although both shortest path length and global efficiency are describing information transfer speed, global efficiency may be especially meaningful when considering the disconnected networks which have nodes don’t have any connection to other nodes in the graph, as paths between disconnected nodes are defined to have infinite path length and this situation can be easily characterized by zero efficiency [4]. Unlike global efficiency, the E_loc_ measures how efficient communication is among the first neighbors of a given node when it is removed, reflecting the fault tolerant of the system [7]. Generally, small-world networks have high E_g_ and E_loc_ at the same time. C_p_ describes the ability for functional segregation and efficiency of local information transfer. The clustering coefficient C_p_ at node p is a fraction of the number of existing connections between the neighbors of the node divided by the number of all possible connections of the graph. The E_loc_ plays a role similar to the C_p_. A network is a small-world network if it has a similar path length but greater clustering of nodes than an equivalent random graph which has the same number of nodes and edges. To quantify small‐world properties, the C_p_ and L_p_ of the network were compared with C_random_ and L_random_, the means for 100 matched random networks with the same number of nodes, edges and degree distribution as the real network [8]. Both normalized C_p_ and normalized L_p_ have the same meaning as C_p_ and L_p_. The σ metric is defined by the ratio between γ and λ. A small‐world network is defined with small worldness σ = γ/λ > 1, which fulfills the conditions of γ (normalized C_p_ = C_p_/C_random_) > 1 and λ (normalized L_p_ = L_p_/L_random_) ≈ 1 [9].

## Machine learning

All machine learning methods were based on the python 3.6.9 environment and the scikit-learn library 0.24.1 (<https://sklearn.org/>) [10]. The upper triangle WM network matrices of patients with ALS were used as the raw features for classification i.e., 4005 connection values for each pair of brain regions. To avoid the ‘curse of dimensionality’, principal component analysis (PCA) was used to reduce the dimensions of the feature space: to increase the signal-to-noise ratio, only the PCs which contain 80% variance of the raw feature space were retained (Fig. S1).

After converting the connections to PCs, the linear kernel support vector machine (SVM) is used to classify the patients with fast and slow progression rate, as follows: first, the model maps the input data from the training set to the feature space using a set of mathematical functions known as kernels. Here, a linear kernel was preferred to a nonlinear kernel to minimize the risk of overfitting and the weights of the linear kernel SVM were thought more interpretable compared with other non-linear kernels. In this feature space, the model learns the optimum separation surface that maximizes the margin between different classes. In our case the linear SVM has one hyperparameter (the soft margin parameter C), which affects the model’s training by controlling the trade-off between reducing training errors and increasing the separation margin. Once the separation surface is determined, it can be used to predict the class of new unseen observations.

To obtain a reliable estimate of the performance of the models, we used a 10-fold stratified cross-validation scheme i.e., outer loop. In this scheme the participants were divided into 10 non-overlapping partitions, each with the same proportion of patients and healthy controls. In each one of the ten iterations of the cross-validation, nine partitions were used as the training set to train the SVM model, and then the trained model was used to obtain predictions in the remaining partition. These predictions were used to calculate the performance metrics (balanced accuracy, specificity, and sensitivity), and since the test set was not part of the training process, the resulting values were unbiased. The reported performance in each case is the mean value across the cross-validation iterations. Finally, the statistical significance was estimated using the permutation method (1,000 permutations).

In each iteration of the cross-validation, we also performed a nested cross-validation inside the training set (i.e., 10-fold stratified nested cross-validation) to select the optimum C value for the SVM, i.e., inner loop. This parameter was optimized by performing a grid search in the following range of values: C = 10^-3^, 10^-2^, 10^-1^, 1, 10, 10^2^, 10^3^, 10^4^. After selecting the best C value based on the balanced accuracy, an SVM was trained using the whole training set and used to assess performance on the test set. Note that the test set was not used during this hyperparameter search, to avoid biased results.

## References:

1. Sarwar T, Ramamohanarao K, Zalesky A. Mapping connectomes with diffusion MRI: deterministic or probabilistic tractography? Magn Reson Med. 2019;81(2):1368-84.

2. Mori S, Kaufmann WE, Davatzikos C, Stieltjes B, Amodei L, Fredericksen K, et al. Imaging cortical association tracts in the human brain using diffusion-tensor-based axonal tracking. Magn Reson Med. 2002;47(2):215-23.

3. Mori S, Crain BJ, Chacko VP, van Zijl PC. Three-dimensional tracking of axonal projections in the brain by magnetic resonance imaging. Ann Neurol. 1999;45(2):265-9.

4. Rubinov M, Sporns O. Complex network measures of brain connectivity: uses and interpretations. Neuroimage. 2010;52(3):1059-69.

5. Achard S, Bullmore E. Efficiency and cost of economical brain functional networks. PLoS Comput Biol. 2007;3(2):e17.

6. Freeman LC. A set of measures of centrality based on betweenness. Sociometry. 1977:35-41.

7. Latora V, Marchiori M. Efficient behavior of small-world networks. Phys Rev Lett. 2001;87(19):198701.

8. Gong G, He Y, Concha L, Lebel C, Gross DW, Evans AC, et al. Mapping anatomical connectivity patterns of human cerebral cortex using in vivo diffusion tensor imaging tractography. Cereb Cortex. 2009;19(3):524-36.

9. Watts DJ, Strogatz SH. Collective dynamics of 'small-world' networks. Nature. 1998;393(6684):440-2.

10. Pedregosa F, Varoquaux G, Gramfort A, Michel V, Thirion B, Grisel O, et al. Scikit-learn: Machine Learning in Python. Journal of Machine Learning Research. 2012;12(10):2825-30.
